# Highly Sensitive MoS_2_ Photodetectors Enabled with a Dry-Transferred Transparent Carbon Nanotube Electrode

**DOI:** 10.1021/acsami.2c19917

**Published:** 2023-01-12

**Authors:** Er-Xiong Ding, Peng Liu, Hoon Hahn Yoon, Faisal Ahmed, Mingde Du, Abde Mayeen Shafi, Naveed Mehmood, Esko I. Kauppinen, Zhipei Sun, Harri Lipsanen

**Affiliations:** † Department of Electronics and Nanoengineering, School of Electrical Engineering, Aalto University, EspooFI-02150, Finland; ‡ Department of Applied Physics, School of Science, Aalto University, EspooFI-02150, Finland

**Keywords:** photodetector, MoS_2_, transferred electrode, carbon nanotube film, tunneling

## Abstract

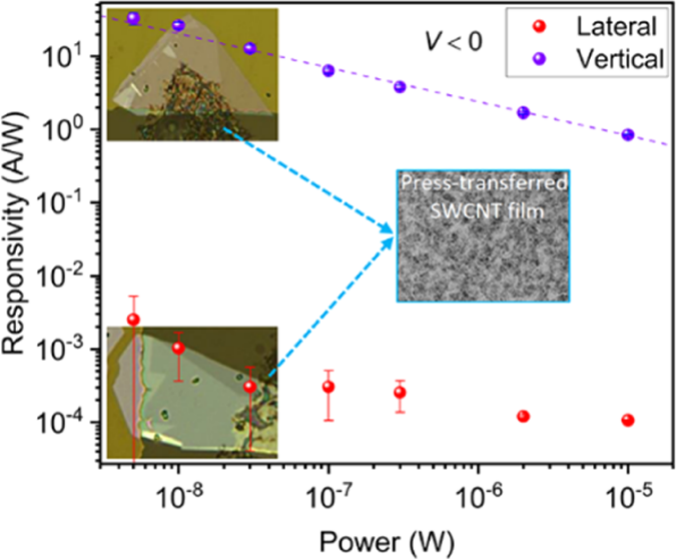

Fabricating electronic and optoelectronic devices by transferring pre-deposited metal electrodes has attracted considerable attention, owing to the improved device performance. However, the pre-deposited metal electrode typically involves complex fabrication procedures. Here, we introduce our facile electrode fabrication process which is free of lithography, lift-off, and reactive ion etching by directly press-transferring a single-walled carbon nanotube (SWCNT) film. We fabricated Schottky diodes for photodetector applications using dry-transferred SWCNT films as the transparent electrode to increase light absorption in photoactive MoS_2_ channels. The MoS_2_ flake vertically stacked with an SWCNT electrode can exhibit excellent photodetection performance with a responsivity of ∼2.01 × 10^3^ A/W and a detectivity of ∼3.2 × 10^12^ Jones. Additionally, we carried out temperature-dependent current–voltage measurement and Fowler–Nordheim (FN) plot analysis to explore the dominant charge transport mechanism. The enhanced photodetection in the vertical configuration is found to be attributed to the FN tunneling and internal photoemission of charge carriers excited from indium tin oxide across the MoS_2_ layer. Our study provides a novel concept of using a photoactive MoS_2_ layer as a tunneling layer itself with a dry-transferred transparent SWCNT electrode for high-performance and energy-efficient optoelectronic devices.

## Introduction

Two-dimensional (2D) materials have attracted increasing attention due to their extraordinary electrical and optoelectronic properties.^1^ Among 2D semiconducting transition-metal dichalcogenides, MoS_2_ is widely studied due to its extremely large optical absorption in the visible spectrum^2^ together with the features of tunable band gap and self-passivated surface, leading to its application in optoelectronic devices.^3−6^ The electrodes of optoelectronic devices are typically deposited by an invasive electron beam or thermal evaporation process in a high vacuum system, which induces defects in the underlying semiconductor layer, and the process is of relatively high cost. Alternatively, recent studies have demonstrated that the electrodes can also be fabricated by the polymer-assisted transfer of pre-deposited metal electrodes to the semiconductor layer from a sacrificial substrate,^7−9^ which improves the device performance by preventing interface damage. Nevertheless, the metal-transfer process reported so far is complicated and cost-inefficient since it requires a complete transfer of uniform metal layers without cracks.

Owing to the distinguished electrical conductivity, high optical transparency, and excellent flexibility/stretchability, single-walled carbon nanotubes (SWCNTs) as a device component would significantly improve the performance of various optoelectronic devices including solar cells, light-emitting diodes, and photodetectors.^10,11^ Press transfer of SWCNTs from a membrane filter to any smooth substrate has been validated as an efficient way to investigate the electrical properties of SWCNTs.^12,13^ The dry-transferred SWCNTs have been integrated into macroscale optoelectronics to elevate the performance.^14−16^ For instance, Jang et al.^16^ reported that the CNT film as the electrode in organic photodetectors could suppress dark current and result in high detectivity accordingly. A highly transparent electrode has been predicted to increase the power conversion efficiency of photovoltaics^8^ and the responsivity of photodetectors.^17^ Recently, it has been reported that the gate modulation of 2D material-based electronic and optoelectronic devices can be enhanced with isolated SWCNTs^18−20^ and the SWCNTs in dispersion.^21,22^ Compared to the 2D/2D material heterostructure, a stronger electric field could exist at the line–plane interface of MoS_2_/SWCNTs, which facilitates the charge carriers to pass through the interface.^23^ However, the 2D material-based devices with the press-transferred SWCNT film used as either an electrode or a heterostructure component have not been realized so far. Our press-transfer method of the SWCNT film is free from lithography, lift-off, and reactive ion etching, thus avoiding polymer residues and retaining the intrinsic mobilities of 2D materials and SWCNTs. Furthermore, the massive chemical synthesis and large-area transfer of SWCNTs have been relatively mature, so the SWCNT-transferred method is promising for practical application in the near future. Additionally, Choi et al.^24^ reported that a lateral heterostructure has distinct advantages over the vertical counterpart which brings in considerable junction resistance between the vertically stacked materials. On the other hand, other reports argued that the devices in a vertically stacked geometry exhibit high current density in a transistor^25^ and fast photoresponse in a photodetector.^26^ Despite this controversy, a systematic investigation of the device configuration, that is, lateral or vertical architecture of the electrode and 2D material channel, to explore the dominant mechanism of charge carrier transport, has rarely been investigated yet.

In this study, we report an easy-accessed dry transfer of transparent SWCNT film onto MoS_2_ as one of the electrodes for the fabrication of high-performance and energy-efficient photodetectors. The SWCNT film is deterministically press-transferred onto MoS_2_ with a micromanipulator under an optical microscope. We fabricated multilayer MoS_2_-based photodetectors both in lateral and vertical configurations. Temperature-dependent current–voltage (*I*–*V*) characteristics and theoretical tunneling models were employed to extract the Schottky barrier height and figure out the dominant charge transport mechanism, respectively. The photodetection performance of the vertically stacked SWCNT/MoS_2_/indium tin oxide (ITO) structure is superior to that of the lateral one due to the Fowler–Nordheim tunneling (FNT) and internal photoemission (IPE) of charge carriers excited from ITO across the MoS_2_ layer in the vertical configuration.

## Results and Discussion

Before the systematic investigation of the influence of device configuration, we investigated the thickness dependency of MoS_2_ flakes to select the flake with an appropriate thickness for further study. We first fabricated devices using thin (∼10 nm thick) and thick (∼52 nm thick) MoS_2_ flakes in a lateral configuration (metal–semiconductor–metal). ITO and SWCNT films were utilized as two electrodes, and mechanically exfoliated multilayer MoS_2_ acts as the semiconductor channel. An important parameter in characterizing diode behavior is the rectification ratio (RR) which is defined as the ratio of forward current to reverse current at the same bias magnitude. As seen from the *I*–*V* characteristics, the thin flake shows about 250 times higher RR value than that of the thicker one under a laser power of 50 μW (Figure S1). In 2D channel devices, a higher RR value is an indicator of higher Schottky barrier height, revealing that the electron affinity of MoS_2_ is dependent on the thickness, which has been confirmed as well in other reports.^27,28^ For further study, we fixed the thickness of MoS_2_ in the range of 5–12 nm to exclude the contribution of high reverse current from thick flakes because of the low Schottky barrier, which will be discussed in detail below.

**Figure 1:**
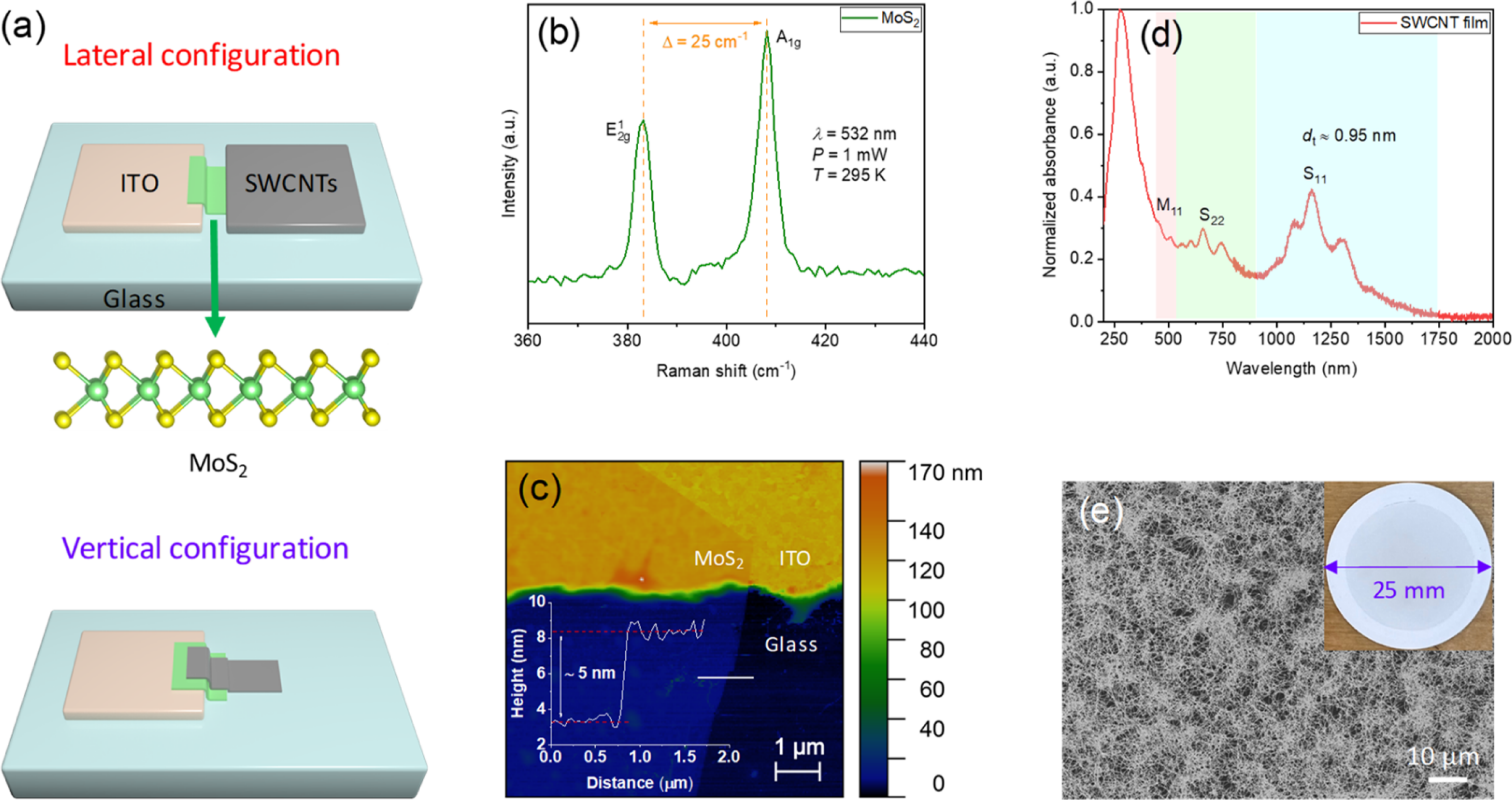
Device schematics and material characterizations. (a) Schematic illustration of the configurations of lateral (top) and vertical (bottom) devices. (b,c) Raman spectrum and AFM image of the lateral device, respectively. The inset in (c) shows the height profile of the MoS_2_ flake edge. (d,e) Optical absorption spectrum and SEM image of SWCNT films, respectively. The inset in (e) shows a photograph of an SWCNT film on a membrane filter.

To figure out the optimal device configuration for the photodetection, we fabricated the devices in both lateral and vertical (metal/semiconductor/metal) geometries (Figure 1a). The Raman spectrum of MoS_2_ displays in-plane vibration of molybdenum and sulfur atoms, *E*_2g_^1^, and out-of-plane vibration of sulfur atoms, *A*_1g_, at 383.2 and 408.2 cm^–1^, respectively (Figure 1b). The frequency separation of the two characteristic peaks is 25 cm^–1^, suggesting that the MoS_2_ flake is multilayered.^29^ The exact thickness of the MoS_2_ flake was confirmed to be ∼5 nm with an atomic force microscope (AFM) (Figure 1c), indicating that the flake roughly contains seven layers. The optical absorption spectrum of SWCNTs clearly shows their three main characteristic peaks, that is, van Hove singularity transitions *E*_S_^11^, *E*_S_^22^ of semiconducting SWCNTs, and *E*_M_^11^ of the metallic counterpart (Figure 1d).^30^ The mean diameter (*d*_t_) of the SWCNTs was estimated to be ∼0.95 nm from the position of the S_11_ peak in the optical absorption spectrum.^13^ The SWCNT film has an optical transparency of 75.9% (Figure S2a), which is ascribed to the porous morphology of the SWCNT network, as shown in the scanning electron microscopy (SEM) image (Figure 1e). Additionally, the SWCNT film exhibits a reasonable pristine sheet resistance value close to 1000 Ω/sq (Figure S2a) and has good quality, which can be concluded from the relatively low disorder-induced band^31^ (Figure S2b). We also presented the Raman spectrum of the stacked heterostructure of SWCNTs and MoS_2_ (Figure S3). The Raman peaks of SWCNTs and MoS_2_ can be observed nearly the same as in the spectra of each individual material.

**Figure 2:**
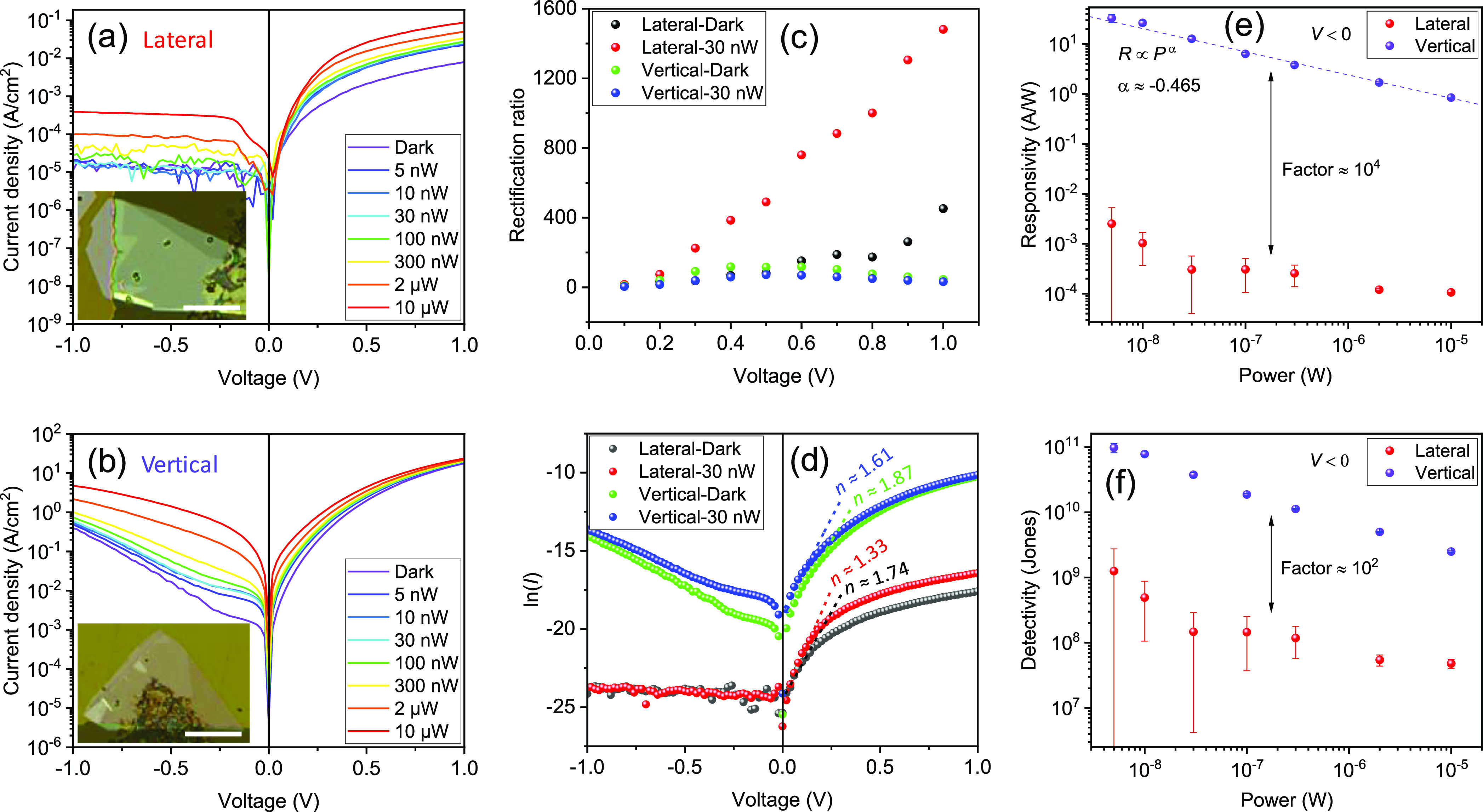
Comparison of the electrical and optoelectronic performance of the diodes in two configurations. (a,b) Semi-logarithmic plots of current density against voltage curves of the devices in lateral and vertical geometries, respectively. Laser is illuminated on the MoS_2_ channel and SWCNT top electrode in (a,b), respectively. The insets show optical microscopy images of two devices. Scale bars, 10 μm. The thickness of MoS_2_ in the two devices is ∼5 nm. ImageJ software was utilized to calculate the active area (typically in the range of 200–300 μm^2^) of a device. (c,d) Plots of RR and ln(*I*) as a function of applied voltage, respectively. The values of *n* in (d) are the extracted ideality factors. (e,f) Responsivity and detectivity as a function of laser power, respectively. The dashed line in (e) is a linear fitting of the responsivity values.

To evaluate the electrical and optoelectronic performance of the devices, we recorded the *I*–*V* curves of the devices under both dark and illuminated conditions. The focused continuous wave 532 nm laser was selected as the light source in this work. As the devices work in the Schottky diode mode, we mainly focused on the device behavior under reverse bias unless specified elsewhere. The vertical device exhibits nearly 4 orders of magnitude higher current density than that of the lateral one (Figure 2a,b), which can be easily attributed to the nanometer (∼5 nm) level carrier transport path compared to the micrometer (∼16 μm) long transport in the lateral one. The lateral device is bulk-limited where the charge transport is governed by the in-plane conductivity of ∼16 μm-long MoS_2_ channel, which is significantly longer than the diffusion distance of the charge carriers in MoS_2_ (0.1–0.2 μm^32,33^). Thus, a larger number of photo-excited carriers can be scattered, trapped, and recombined in the channel before being collected at the electrodes. As for the vertical device, the large effective junction area between two electrodes and the sandwiched semiconductor would efficiently harvest photons, producing a considerable amount of photo-excited charge carriers. The large junction area also facilitates the extraction and transport of charge carriers in the MoS_2_ channel, leading to a high current density. On the other hand, the high reverse current in the vertical device results in low RR values. The RR value of the vertical device is only 32.5 at *V* = ±1 V under 30 nW laser illumination, while the lateral structure gives a RR value up to 1.48 × 10^3^ under the same conditions (Figure 2c). Since MoS_2_ with the same thickness is integrated into two structures, the Schottky barrier height should in principle be the same but it is not the case in reality (discussed in detail below). Thus, we would assume that tunneling is the dominant origin of the observed high reverse current in the vertical device with a ∼5 nm-long channel.

We also extracted the ideality factor to know the interface contact quality of the diodes. For a non-ideal diode, the current through the diode as a function of voltage can be expressed with the Shockley diode equation^34^
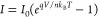
1where *I*_0_, *q*, *n*, *k*_B_, and *T* are reverse saturation current, elementary charge, ideality factor, Boltzmann constant, and temperature, respectively. As the term “–1” is negligible at high voltages (>0.05–0.1 V), then taking the natural logarithm of both sides of the equation generates
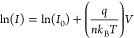
2When ln(*I*) is plotted against *V*, the ideality factor *n* can be calculated from the slope of . An ideal diode has an ideality factor of 1, which implies that the net current through the diode is induced by carrier diffusion instead of recombination. The existence of some defects or impurities which act as trap sites can lead to the recombination of charge carriers at the junction, generating an ideality factor higher than 1. The ideality factors of the diodes under dark conditions are close to 2 (Figure 2d), suggesting that there exist some charge trapping states or recombination centers at the junction area. However, under 30 nW laser illumination, the ideality factors of the diodes are approaching 1 as the recombination probability is decreased upon light illumination. Some photo-excited charge carriers have enough energy to overcome their trapping by the defects or impurities, resulting in low recombination current and ideality factors accordingly.^3^ The lateral device presents lower ideality factors in both dark and illuminated conditions compared with the vertical one, which is consistent with the results shown in Figure 2c.

We next assessed the devices with the figure of merits (i.e., responsivity and detectivity) for photodetection. The responsivity (*R*) of a photodetector is the ratio of photocurrent to effective power
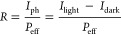
3where *I*_ph_ is the photocurrent, *I*_light_ and *I*_dark_ are the currents under light and dark conditions, respectively, and *P*_eff_ is the power that is effectively illuminated on the active area of a device. The detectivity (D*) of a photodetector is used to evaluate the sensitivity of a detector and is defined by the formula
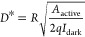
4where *R*, *A*_active_, *q*, and *I*_dark_ are the responsivity, active area of a device, elementary charge, and dark current, respectively. The average responsivity value of the vertical device is approximately 10^4^ times higher than that of the lateral one (Figure 2e), showing a maximum responsivity of 33 A/W at *V* = −1 V under 5 nW laser illumination. The responsivity values of the photodetectors are decreasing with increasing laser power since the photocurrents are gradually saturating on account of the screening effect under high-power illumination.^35,36^ Furthermore, the vertical device is nearly 10^2^ times more sensitive than the lateral one (Figure 2f), exhibiting a maximum detectivity of 9.7 × 10^10^ Jones at *V* = −1 V under 5 nW laser illumination. Except for the aforementioned fast charge separation and transport in the vertical device, the high transparency of top and bottom electrodes is another major contributor to the outstanding photodetection performance since the sandwiched MoS_2_ layer can absorb more light in the vertical device as compared with the opaque photodetectors. In addition, optical switching response of the devices was checked. Thanks to the diode structure, the lateral device working under reverse bias has a higher response speed (Figure S4).^37^ The depletion width of the lateral device increases under reverse bias, which decreases the junction capacitance and facilitates the transport of charge carriers accordingly. Thus, the transit time of charge carriers is reduced, elevating the optical response speed of the lateral device.^38,39^ Therefore, based on the above results, we can conclude that a device in the vertical configuration is more suitable for photodetection, while the one in lateral geometry can be potentially utilized as a rectifier.^40^

**Figure 3:**
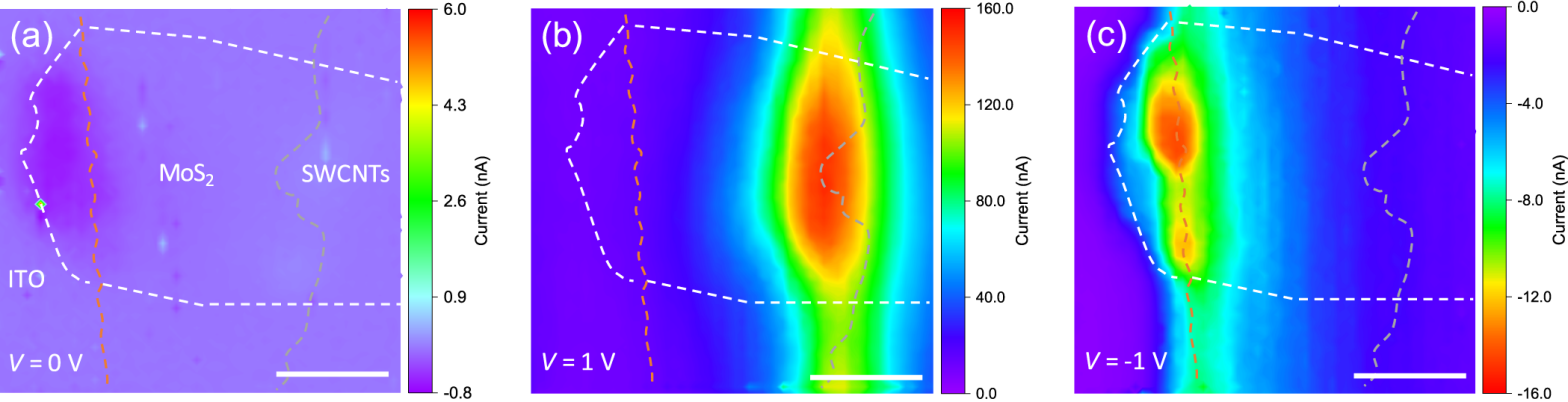
Photocurrent mapping of the lateral device. The bias voltages for (a–c) are 0, 1, and −1 V, respectively. The red, white, and gray dashed lines outline the locations of ITO, MoS_2_, and SWCNTs, respectively. Laser power, 1 μW. Scale bars, 10 μm.

To further probe the spatial distribution of photocurrent, we carried out photocurrent mapping of the lateral device. The photocurrent mapping obtained at zero bias voltage displays a higher current at the junction between ITO and MoS_2_ than that at the SWCNT side (Figure 3a), signifying a higher Schottky barrier at the ITO side. This is an indicator for us to sketch the band diagrams, as presented below. It is clear that the photocurrent at forward bias (*V* > 0) is mainly generated at the junction of MoS_2_ and SWCNTs (Figure 3b). The spread of current along the interface is attributed to the unevenly distributed SWCNTs at the edge of the SWCNT film, which is inevitable when a doctor blade is used for cutting the film. The high forward current is mainly originated from a low barrier and could be caused by the thermionic emission of the electrons injected from SWCNTs. Furthermore, the electron–hole pairs generated at the junction can be efficiently separated by the built-in potential and contribute to the observed current. Additionally, as more than two-thirds of the SWCNTs are p-type semiconductors, which has been confirmed in our previous work,^41^ the p–n heterojunction formed at the MoS_2_/SWCNT interface is another contributor to the photocurrent.^21^ In contrast, the junction between ITO and MoS_2_ is responsible for the low reverse current, which is possibly initiated by thermal-assisted tunneling due to the existence of a high Schottky barrier (Figure 3c).

**Figure 4:**
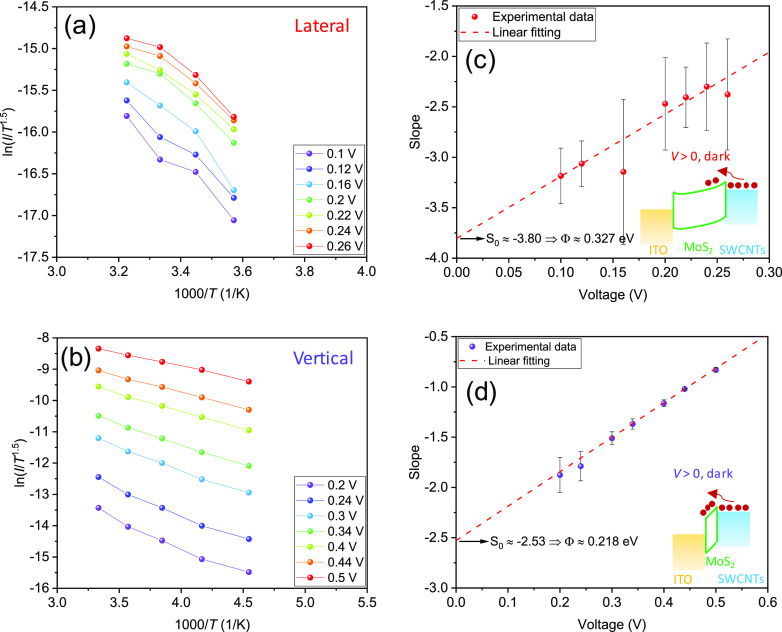
Temperature-dependent results under the dark condition for the extraction of Schottky barrier height. (a,b) Arrhenius plots of  vs 1000/*T* at different voltages. Only forward bias was considered. The temperature ranges of 280–310 and 220–300 K were selected in (a,b), respectively, for linear fitting. (c,d) Slope values extracted by linear fitting the Arrhenius plots in (a,b), respectively, as a function of applied voltage. The insets show the corresponding band diagrams to illustrate the extracted barrier heights under dark conditions.

To gain insights into the conduction mechanism, we separately analyzed the charge transport behavior at forward and reverse biases. First, we recorded the temperature-dependent *I*–*V* curves of the two devices working at forward bias (Figure S5). The current increases with the temperature in both devices, indicating that the charge carrier transport through the Schottky diode is dominated by thermionic emission at high temperatures, while tunneling is expected to play a governing role at lower temperatures (Figure S6). The charge carriers may not have enough energy at low temperatures to surmount the barrier height, and thus, the transport is governed by tunneling across the Schottky barrier. The thermionic emission current flowing through the 2D materials can be expressed with the following equation^42,43^
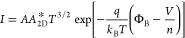
5where *A*, *A*_2D_^*^, *T*, *q*, *k*_B_, Φ_B_, and *n* are the active area of a device, 2D equivalent Richardson constant, temperature, elementary charge, Boltzmann constant, Schottky barrier height, and ideality factor. Notably, the reduced power law *T*^3/2^ is employed here as the channel is a 2D semiconductor.^42^ Then, by taking the natural logarithm of both sides, eq 5 can be re-organized to
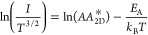
6where  is the activation energy. Then, the Φ_B_ can be calculated from the slope obtained by linearly fitting the Arrhenius plot of  versus 1000/*T* (Figure 4a,b). The slope from each fitting is then plotted as a function of forward bias. The Schottky barrier height Φ_B_ can be finally calculated from the intercept (*S*_0_, where *V* = 0) by following the formula  (Figure 4c,d). The Φ_B_ of the vertical device is smaller than that of the lateral one, which also leads to a higher forward current in the former structure (Figure 2a,b). Lighter band bending in the vertical case because of large-area intimate contact might account for the obtained lower barrier height. In contrast, since the contact area of the lateral device is small, band bending may occur more significantly by a localized electric field when a voltage of the same magnitude is applied.^44^ As illustrated in the band diagrams (the insets in Figure 4c,d), the low Schottky barrier extracted here comes from the junction between MoS_2_ and SWCNTs, which explains the observed high current in the photocurrent mapping in Figure 3b. The obtained low Schottky barrier heights of 0.2–0.3 eV indicate quasi-Ohmic contact between MoS_2_ and SWCNTs.^45^

We then incorporated thicker (∼10 nm) MoS_2_ into the devices of the two configurations (Figure S7a,b) to compare their temperature-dependent trends with those observed with the devices using 5 nm thick MoS_2_ (Figure 4). We confirm that the charge carrier transport of the devices consisting of 10 nm thick MoS_2_ is also dominated by thermionic emission under forward bias at room temperature (Figure S7c,d). Both devices in lateral and vertical configurations exhibit lower barrier heights than those using thinner flakes (Figure S7e,f). This verifies the conjecture mentioned above that a thicker MoS_2_ flake will result in a lower Schottky barrier and RR. A thicker MoS_2_ flake can absorb more light owing to the increased number of layers and narrowed band gap. Inspired by this thickness-dependent behavior, we fabricated another vertical device using ∼155 nm-thick MoS_2_ to further improve the photodetection performance. The photodetector demonstrates an ultrahigh responsivity of up to 2008.3 A/W and an excellent detectivity value of 3.2 × 10^12^ Jones simultaneously at *V* = 1 V with 10 nW laser illumination (Figure S8). The responsivity value is much higher than those of reported MoS_2_-based photodetectors while the detectivity is still competitive^4,46−48^ (Table S1). Even at a low bias voltage of 0.1 V, the device presents an excellent responsivity of 70.3 A/W and a high detectivity of 7.7 × 10^11^ Jones, signifying the potential for self-powered photodetectors. The extraordinary optoelectronic performance is attributed to efficient light absorption and effective carrier injection due to the usage of a thick MoS_2_ flake. It should be noted that no gate electrode was applied to the photodetectors investigated in this work, meaning that our devices have low power dissipation.

**Figure 5:**
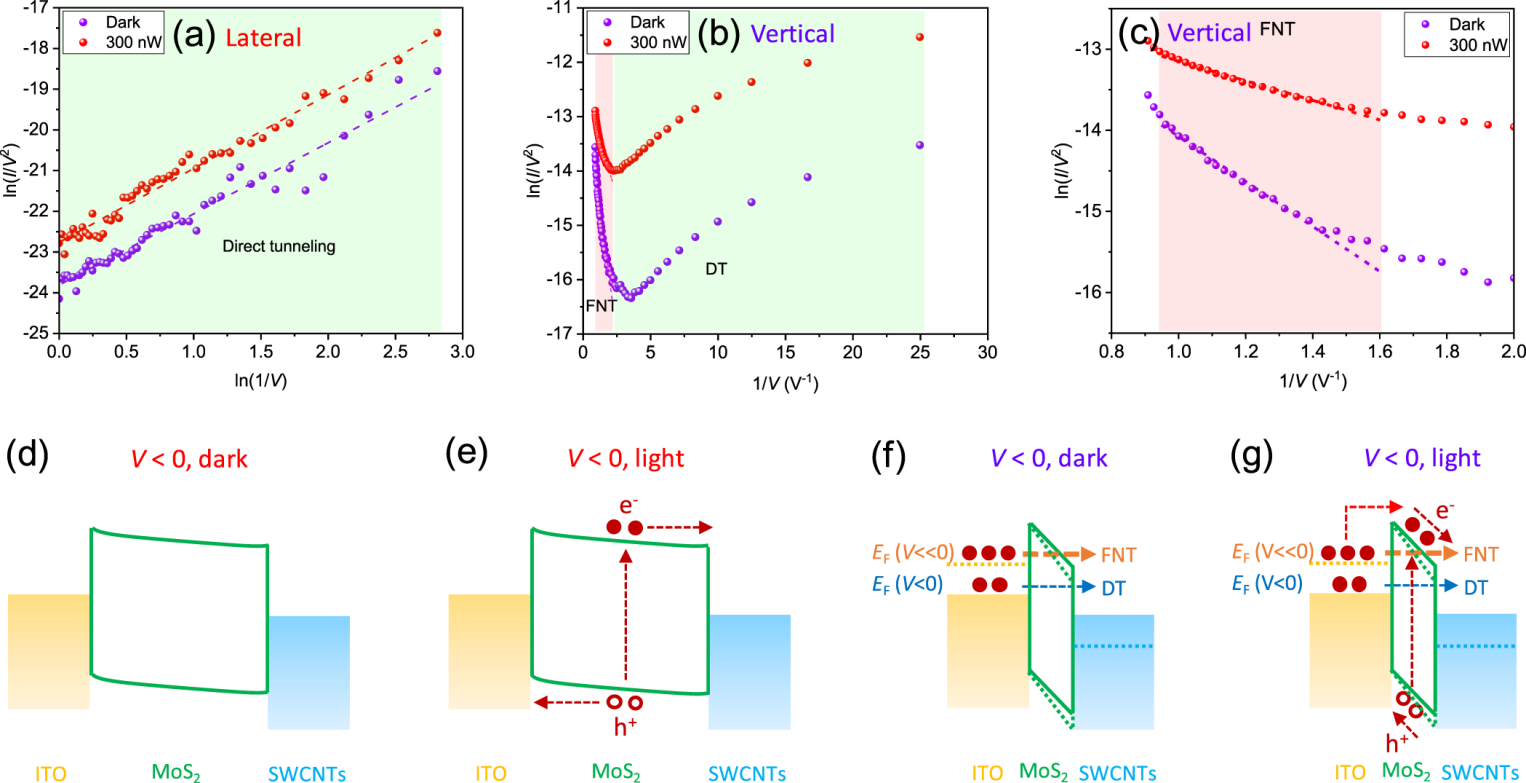
Tunneling plots and band diagrams of the devices under reverse bias. (a) Plots of  vs ln(1/*V*) of the lateral device. The dashed lines are the linear fitting of data points. (b) Plots of  vs 1/*V* of the vertical device. (c) Zoomed-in view of the FNT region marked in pink in (b). (d,e) Band diagrams of the lateral device working under dark and light conditions, respectively. (f,g) Band diagrams of the vertical device working under dark and light conditions, respectively. *E*_F_ (*V* < 0) and *E*_F_ (*V* ≪ 0) represent the Fermi level positions at low and high reverse bias voltages, respectively.

Then, we turn to the origin of the charge carrier transport at reverse bias. As we suggested earlier, the charge transport at reverse bias might be dominated by tunneling. Thus, we adopted direct tunneling (DT) and FNT models^49−51^ to fit the *I*–*V* curves at reverse bias. DT and FNT are expressed using the following equations
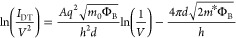
7
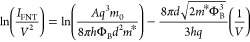
8where *A*, *q*, *m*_0_, Φ_B_, *h*, *d*, and m*(0.45 *m*_0_)^44^ are the junction area, elementary charge, free electron mass, barrier height, Planck constant, the thickness of MoS_2_ flake, and effective electron mass, respectively. The current is induced by DT if the plot of  versus ln(1/*V*) has a positive slope and obeys a linear fitting regime, while the FNT can be recognized from the plot of  versus 1/*V* which displays a negative and linear slope. The carrier conduction in the lateral device is clearly dominated by DT throughout the whole reverse bias range at both dark and illuminated conditions (Figure 5a). Interestingly, the carrier transport in the vertical device working at low voltages is caused by DT (Figure 5b) while the FNT governs the conduction at high voltages (Figure 5c). The shift in the origin of charge carrier injection with the variation in the voltage magnitude has also been observed elsewhere.^49,52^ The minimum transition voltage from DT to the FNT is around 0.28 V. In addition, the barrier height for the FNT was calculated to be ∼0.75 eV by taking the slope value extracted from the fitting result. The FNT is known to make a great contribution to the total current when the barrier height and width are small under high bias voltages.^51−53^

Based on the photocurrent mapping and the fitting results with tunneling models, we present the band diagrams of the devices working under reverse bias in Figure 5d–g for more detailed interpretation. Owing to the high and wide Schottky barrier at the ITO side, only DT can occur in the lateral device working under dark conditions (Figure 5d). With light illumination on the MoS_2_ channel, photo-generated charge carriers can be collected at the electrodes due to the built-in electrical field at the junctions (Figure 5e). The total low current is the origin of the small reverse current, as shown in Figure 2a. As for the vertical device, DT is the dominant transport of charge carriers at low voltages as the field-induced band bending is moderate (Figure 5f). When a higher voltage is applied, the potential barrier at the ITO/MoS_2_ interface shrinks, and the barrier shape changes to triangular (thin) from trapezoidal (wide) accompanied by a decrease in the barrier height.^44,49,53^ Thus, the carriers can tunnel through the triangular barrier via the FNT, resulting in a large reverse current. Moreover, when the vertical device is illuminated from the SWCNT top electrode (Figure 5g), light can penetrate through the ultrathin MoS_2_ layer to the ITO bottom electrode, resulting in the IPE of electrons from the ITO side across the low Schottky barrier. This process benefits from the transparency of both SWCNT top electrodes and MoS_2_ thin flakes. Therefore, the FNT of charge carriers excited from the ITO side through the MoS_2_ barrier layer and the IPE of electrons from the ITO side are the dominant conduction mechanisms of the observed high reverse current in the vertical device, as displayed in Figure 2b. Besides the dominant conduction mechanism, there are several external factors that can affect the reverse current. An obvious factor is temperature. A lower temperature results in the lower reverse current (Figures S5, S7). The thickness, quality, and doping concentration of MoS_2_ also affect the reverse current. Both thinner flake (Figure S1) and better material quality with small number of defects can lead to lower reverse current. The MoS_2_ thickness and doping concentration have been extensively investigated in a vertical device geometry elsewhere.^44^ Thus, one needs to realize that those factors should be consistent for a fair comparison.

## Conclusions

In summary, we demonstrated high-performance MoS_2_-based Schottky photodiodes with press-transferred transparent SWCNT films as the electrode. We found that a device in the lateral configuration using a thin MoS_2_ layer shows excellent rectifying behavior. Sandwiching thick MoS_2_ flake between two transparent electrodes (ITO and SWCNTs) enables outstanding photodetection performance due to efficient light absorption and effective carrier separation, maximizing both responsivity and detectivity. Additionally, temperature-dependent electrical characteristics indicate that the effective Schottky barrier height formed at the SWCNT/MoS_2_ interface is higher in the lateral configuration than that in the vertical one. The superior photodetection performance in the vertical device is ascribed to the FNT and IPE of charge carriers excited from ITO across the MoS_2_ barrier layer. Our idea of using a photoactive MoS_2_ layer as a tunneling layer with a dry-transferred transparent SWCNT electrode paves a high-performance and cost-effective way to develop 2D material-based transparent optoelectronic devices for wearable applications.

## Experimental Section

### Materials and Device Fabrication

Pre-patterned ITO glass substrates were purchased from Ossila, UK and used as received. The sheet resistance of 100 nm thick ITO films is around 20 Ω/sq. MoS_2_ flakes were mechanically exfoliated from the bulk crystal (2D Semiconductors, USA) with the Nitto blue tape and were transferred to the edge of ITO films via a polydimethylsiloxane stamp under an optical microscope. SWCNTs were produced in a floating catalyst chemical vapor deposition reactor using isopropanol and ferrocene as the carbon source and catalyst precursor, respectively. The growth parameters are similar to those reported in our previous work.^41^ Then, the SWCNTs were collected downstream of the reactor with a Millipore membrane filter from which the SWCNTs can be easily press-transferred onto any smooth substrate. The SWCNT film has an optical transmittance of 75.9% and a sheet resistance of ∼1000 Ω/sq. A millimeter-sized SWCNT/filter was cut with a doctor blade and stuck onto a glass substrate with the double-sided tape. The glass substrate with SWCNTs facing down was mounted on a micromanipulator and aligned along the MoS_2_ flake under an optical microscope. Then, the SWCNTs can be deterministically transferred onto the MoS_2_ flake by the gentle press and slow lifting processes.

### Optical Measurement

The optical photographs were taken with a Leica DM6 optical microscope. The Raman spectrum of MoS_2_ was acquired with a WITec alpha300 RA+ Raman spectrometer using a 532 nm laser with a power of around 1 mW. Another Raman spectrometer (HORIBA Jobin-Yvon Labram HR 800) was utilized to record the spectra of SWCNTs with four excitation lasers of 488, 514, 633, and 785 nm. The Raman spectra of SWCNTs were averaged based on three separate spectra collected in different areas. To acquire the optical absorption of SWCNTs, the film was press-transferred onto a quartz substrate, and a UV–vis–NIR spectrometer (Agilent Cary 5000) was utilized to collect the spectrum after the subtraction of substrate contribution via positioning another clean quartz substrate in the reference beam path. The optical transmittance value of the SWCNT film was determined at 550 nm from a transmittance spectrum, ranging from 500 to 600 nm.

### Microscopy Measurement

To know the thicknesses of MoS_2_ flakes, a Bruker Dimension Icon AFM operated in a tapping mode was employed for the acquisition of topological images which were later analyzed with Gwyddion software for thickness measurement. To view the surface morphology of the SWCNT film, the film was transferred onto the SiO_2_/Si substrate and densified with ethanol prior to the imaging with an scanning electron microscope (Zeiss Sigma VP) operated at 1 kV using the in-lens detector.

### Electrical and Optoelectronic Measurements

The as-fabricated devices were subjected to electrical and optoelectronic measurements without any further treatment. The devices were fixed to a Linkam LN600-P sample holder on the stage of a SNOM system (WITec alpha300). The current–voltage (*I*–*V*) curves of the devices were recorded with a customized LabVIEW program by applying the voltage with a Keithley 2401 source meter. For the photodetection test, the green laser (532 nm) with adjustable power was selected as the light source and illuminated on the devices through a 20× objective (NA = 0.4, the diameter of the laser spot is around 4.4 μm). The data acquisition of the devices at low temperature was realized in the liquid nitrogen atmosphere with a home-made Linkam stage coupled with an environmental and temperature controller (T96-S).

## Supporting Information Available

MoS_2_ thickness-dependent diode behavior; AFM characterization of MoS_2_ flakes; optical and electrical characterizations of SWCNT films; Raman spectrum of the heterostructure of SWCNTs/MoS_2_; transient photoresponse of the devices; temperature-dependent electrical measurement of the devices; photodetection performance of the device using 155 nm thick MoS_2_; and comparison of the responsivity and detectivity values of MoS_2_-based photodetectors (PDF)
