# Supplementary material for: Highly Sensitive MoS2 Photodetectors Enabled with a Dry-Transferred Transparent Carbon Nanotube Electrode
Source: ACS Appl Mater Interfaces. 2023 Jan 12;15(3):4216–25. doi: 10.1021/acsami.2c19917 (PMC9880956; doi:10.1021/acsami.2c19917)
Supplement: Supplementary file 1 — am2c19917_si_001.pdf [file am2c19917_si_001.pdf]

Highly Sensitive MoS_2 Photodetectors Enabled with a Dry-Transferred Transparent Carbon Nanotube Electrode

Er-Xiong Ding[1,*], Peng Liu[2,1], Hoon Hahn Yoon[1], Faisal Ahmed[1], Mingde Du[1], Abde Mayeen Shafi[1], Naveed Mehmood[1], Esko I. Kauppinen[2], Zhipei Sun[1], and Harri Lipsanen[1,*]

[1]Department of Electronics and Nanoengineering, School of Electrical Engineering, [2]Department of Applied Physics, School of Science, Aalto University, Espoo FI-02150, Finland

[*]Corresponding authors

erxiong.ding@aalto.fi harri.lipsanen@aalto.fi

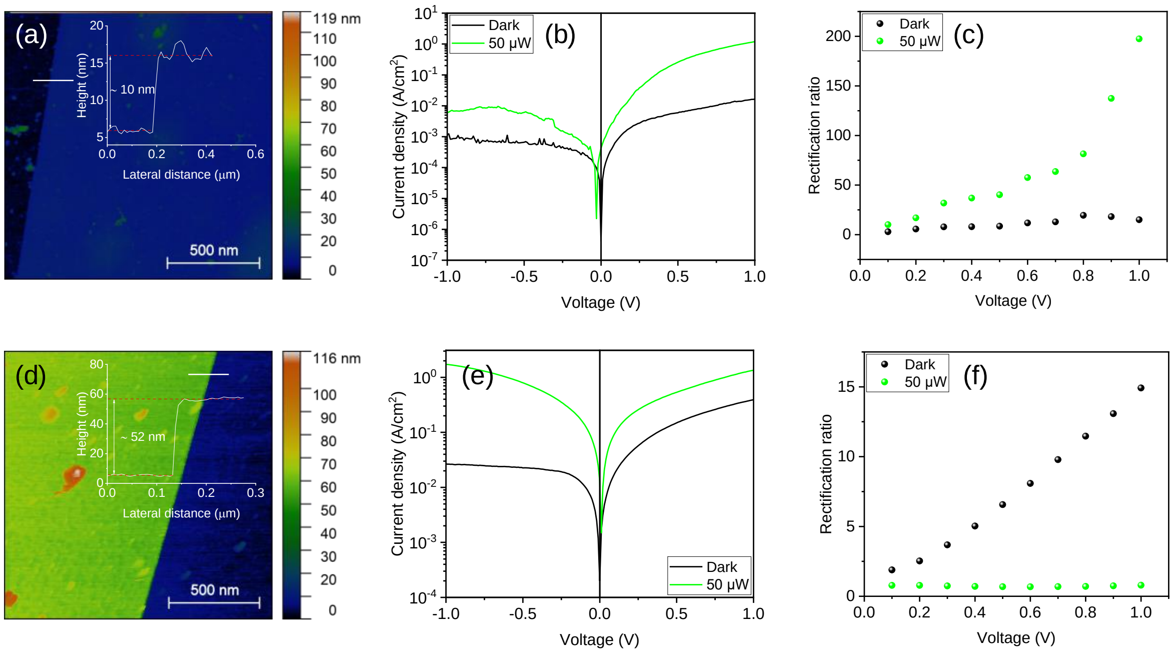

Figure S1. Preliminary study of MoS_2 thickness-dependent diode behavior. (a,d) Atomic force microscopy (AFM) images of thin (ca. 10 nm) and thick (ca. 52 nm) MoS_2 flakes, respectively. The insets display the height profiles of the flakes. (b,e) Plots of current density in log scale versus applied voltage for the lateral device using thin and thick MoS_2 flakes, respectively. (c,f) Plots of rectification ratio extracted from (b) and (e), respectively as a function of applied voltage.

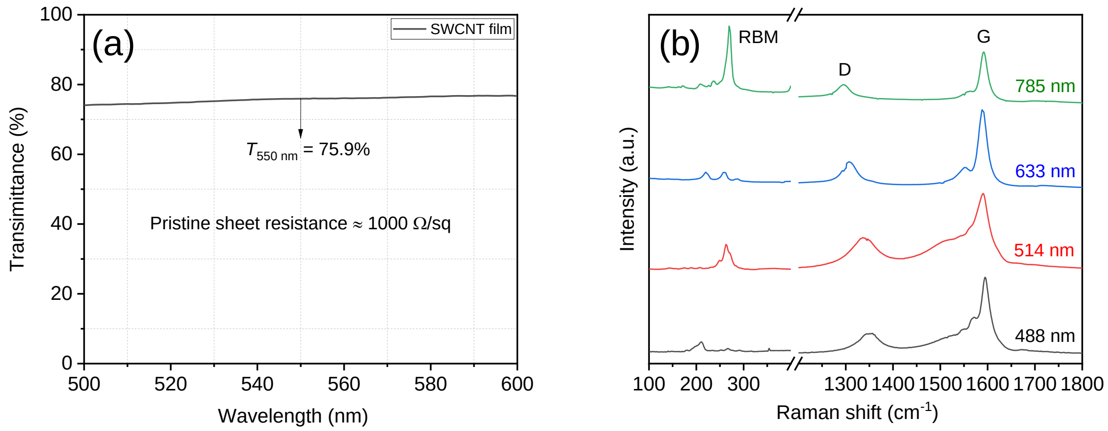

Figure S2. Optical and electrical characterizations of SWCNT film. (a) Optical transmittance spectrum of SWCNT film. The transmittance value was obtained at 550 nm. The sheet resistance of SWCNT film was obtained with a Jandel four-point probe station after the film was press-transferred onto a quartz substrate from a membrane filter. (b) Raman spectra of SWCNTs were acquired with 488, 514, 633, and 785 nm laser excitation. The characteristic peaks originating from graphitic mode (G), disorder-induced mode (D), and radial breathing mode (RBM) are clearly presented. RBM is a signal of SWCNTs.

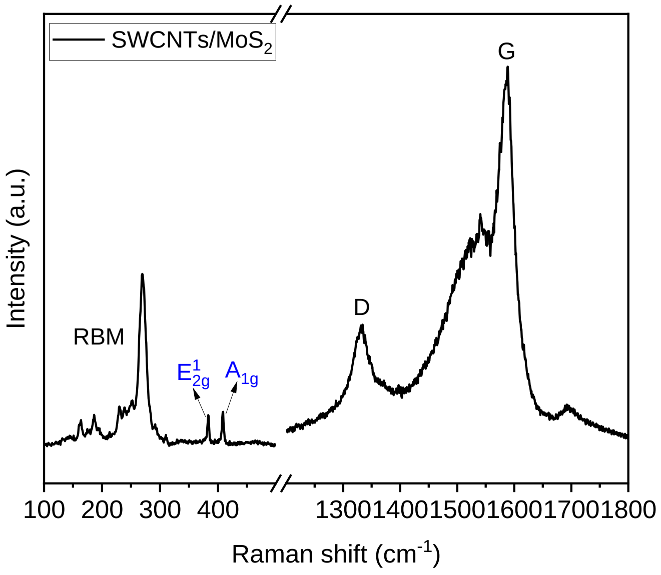

Figure S3. Raman spectrum of the stacked structure of SWCNTs and MoS_2. Laser wavelength, 532 nm, laser power, 1 mW.

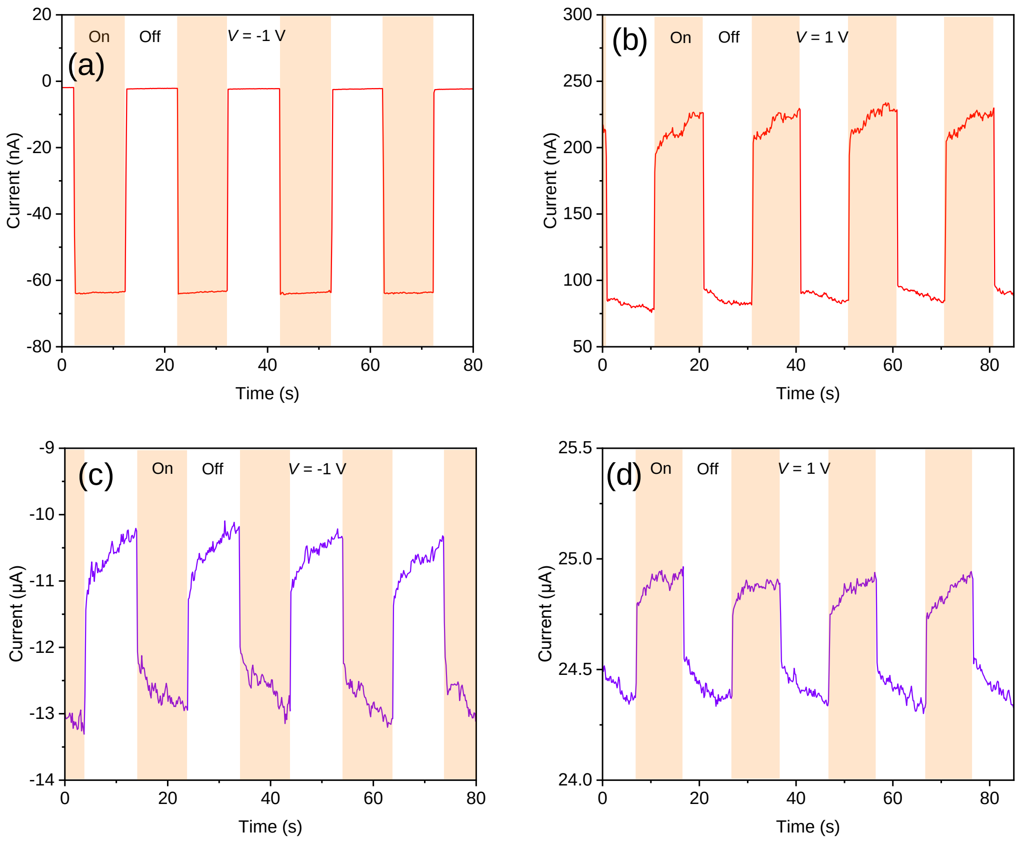

Figure S4. Transient photoresponse of the devices. (a,b) Time-dependent current of the lateral device measured under reverse and forward biases, respectively. (c,d) Time-dependent current of the vertical device measured under reverse and forward biases, respectively. The illumination of 532 nm laser was manually switched on/off for 10 s. Laser power was set to be 2 μW.

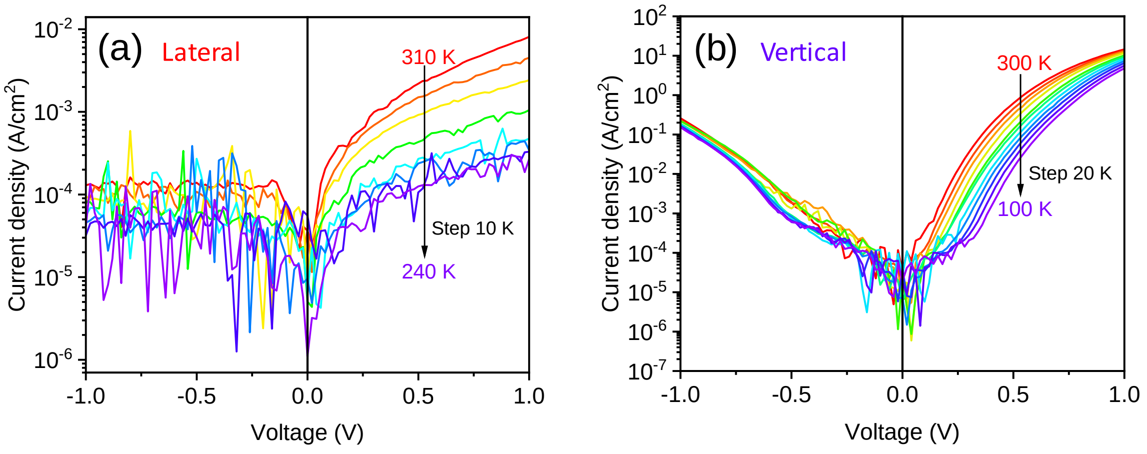

Figure S5. The plots of temperature-dependent current density in log scale versus voltage for (a) lateral device and (b) vertical device using ca. 5 nm-thick MoS_2 flake.

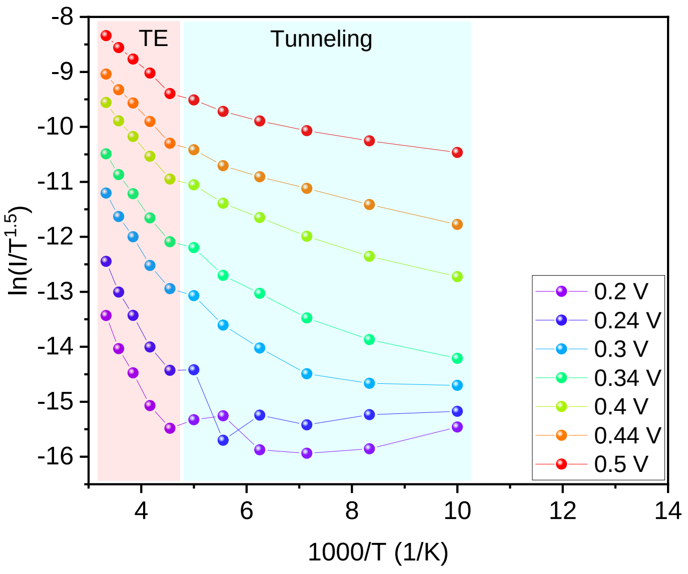

Figure S6. The Arrhenius plots of $\ln\left(I/T^{3/2}\right)$ versus $1000/T$ of the vertical device with ca. 5 nm thick MoS_2 flake at different forward voltages. TE is short for thermionic emission.

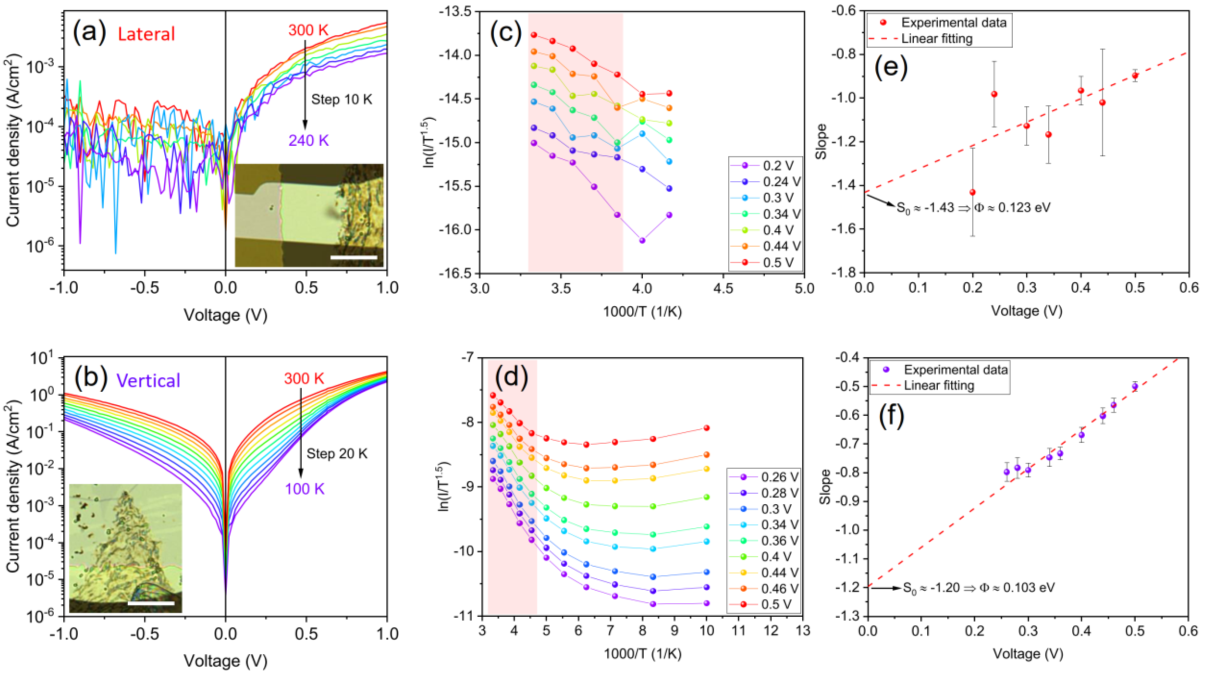

Figure S7. Temperature-dependent electrical measurement of the devices using thick MoS_2 flake. (a,b) Plots of current density in log scale versus voltage at various temperatures for the lateral and vertical devices, respectively. The insets are optical microscopy images of two device structures. Scale bars, 10 μm. The flake thicknesses in (a) and (b) are ca. 10 nm and ca. 12 nm, respectively. (c,d) The Arrhenius plots at different forward voltages. Linear fitting was analyzed in the areas marked in pink. (e,f) Plots of the slope values obtained from the linear fitting of the data in (c) and (d), respectively to extract the Schottky barrier height.

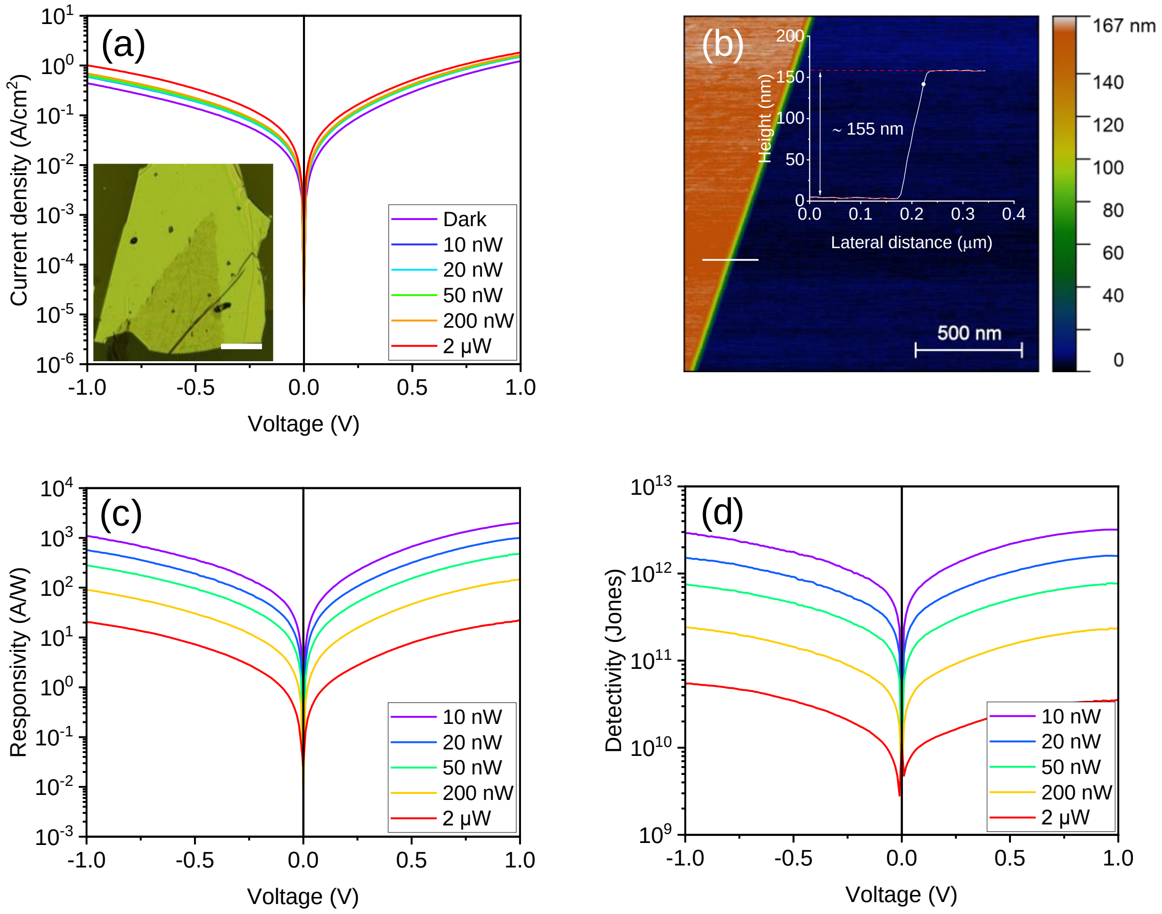

Figure S8. A vertical device using thick (ca. 155 nm) MoS_2 as the semiconductor channel. (a) Laser power-dependent plots of current density as a function of voltage. The inset is an optical microscopy image of the device. Scale bar, 50 μm. (b) AFM image of the thick MoS_2 flake. The inset shows the height profile of the flake. (c,d) The power-dependent responsivity and detectivity values versus the applied voltage, respectively.

Table S1. Comparison of the responsivity and detectivity values of MoS_2-based photodetectors.

**Device structure**	**Measurement condition (λ, P, V_{ds}, V_g)**	**Responsivity (A/W)**	**Detectivity (Jones)**	**Reference**
Ti-Monolayer MoS_2-Ti	550 nm, 80 μW, 1 V, 50 V	0.0075	-	1
Pd/Multilayer MoS_2/Au	532 nm, 1 μW/μm^2, 0.1 V, 0 V	0.01	-	2
Au-Multilayer MoS_2-Au	445 nm, 8 mW, 10 V, 0 V	0.0507	1.55×10^9	3
Ti-Multilayer MoS_2-Ti	633 nm, 50 mW/cm^2, 1 V, -3 V	0.115	3.5×10^{10}	4
Graphene-Monolayer MoS_2-Graphene	532 nm, 4 nW, 1 V, 40 V	2	1.2×10^{13}	5
Ti-Monolayer MoS_2-Ti	532 nm, 0.15 mW/cm^2, 0.1 V, 0 V	99.9	9.4×10^{12}	6
Graphene/Multilayer MoS_2/Graphene	532 nm, 6.37 mW/cm^2, -1 V, 40 V	414	3.2×10^{10}	7
Au-Monolayer MoS_2-Au	561 nm, 0.15 nW, 8 V, -70 V	880	-	8
Cr-Multilayer MoS_2-Cr	635 nm, 1 nW, 5 V, 0 V	2570	2.2×10^{12}	9
SWCNTs/Multilayer MoS_2/ITO	532 nm, 10 nW, -1 V, 0 V	1094.3	2.9×10^{12}	This work
	532 nm, 10 nW, 0.1 V, 0 V	70.3	7.7×10^{11}	
	532 nm, 10 nW, 1 V, 0 V	2008.3	3.2×10^{12}	

Note: Metal-semiconductor-metal represents a device in a lateral configuration, while metal/semiconductor/metal stands for a vertical configuration. "–" means the data is unavailable.

References

(1) Yin, Z.; Li, H.; Li, H.; Jiang, L.; Shi, Y.; Sun, Y.; Lu, G.; Zhang, Q.; Chen, X.; Zhang, H. Single-Layer MoS_2 Phototransistors. *ACS Nano* **2012**, *6*, 74–80.

(2) Li, Z.; Chen, J.; Dhall, R.; Cronin, S. B. Highly Efficient, High Speed Vertical Photodiodes Based on Few-Layer MoS_2. *2D Mater.* **2017**, *4*, 015004.

(3) Xie, Y.; Zhang, B.; Wang, S.; Wang, D.; Wang, A.; Wang, Z.; Yu, H.; Zhang, H.; Chen, Y.; Zhao, M.; Huang, B.; Mei, L.; Wang, J. Ultrabroadband MoS_2 Photodetector with Spectral Response from 445 to 2717 nm. *Adv. Mater.* **2017**, *29*, 1605972.

(4) Choi, W.; Cho, M. Y.; Konar, A.; Lee, J. H.; Cha, G. B.; Hong, S. C.; Kim, S.; Kim, J.; Jena, D.; Joo, J.; Kim, S. High-Detectivity Multilayer MoS_2 Phototransistors with Spectral Response from Ultraviolet to Infrared. *Adv. Mater.* **2012**, *24*, 5832–5836.

(5) Liu, B.; Chen, Y.; You, C.; Liu, Y.; Kong, X.; Li, J.; Li, S.; Deng, W.; Li, Y.; Yan, H.; Zhang, Y. High Performance Photodetector Based on Graphene/MoS_2/Graphene Lateral Heterostrurcture with Schottky Junctions. *J. Alloys Compd.* **2019**, *779*, 140–146.

(6) Li, S.; Chen, X.; Liu, F.; Chen, Y.; Liu, B.; Deng, W.; An, B.; Chu, F.; Zhang, G.; Li, S.; Li, X.; Zhang, Y. Enhanced Performance of a CVD MoS_2 Photodetector by Chemical in Situ n-Type Doping. *ACS Appl. Mater. Interfaces* **2019**, *11*, 11636–11644.

(7) Gao, S.; Wang, Z.; Wang, H.; Meng, F.; Wang, P.; Chen, S.; Zeng, Y.; Zhao, J.; Hu, H.; Cao, R.; Xu, Z.; Guo, Z.; Zhang, H. Graphene/MoS_2/Graphene Vertical Heterostructure-Based Broadband Photodetector with High Performance. *Adv. Mater. Interfaces* **2021**, *8*, 2001730.

(8) Lopez-Sanchez, O.; Lembke, D.; Kayci, M.; Radenovic, A.; Kis, A. Ultrasensitive Photodetectors Based on Monolayer MoS_2. *Nat. Nanotechnol.* **2013**, *8*, 497–501.

(9) Wang, X.; Wang, P.; Wang, J.; Hu, W.; Zhou, X.; Guo, N.; Huang, H.; Sun, S.; Shen, H.; Lin, T.; Tang, M.; Liao, L.; Jiang, A.; Sun, J.; Meng, X.; Chen, X.; Lu, W.; Chu, J. Ultrasensitive and Broadband MoS_2 Photodetector Driven by Ferroelectrics. *Adv. Mater.* **2015**, *27*, 6575–6581.
